# Activin A: a novel urinary biomarker of renal impairment in multiple myeloma

**DOI:** 10.1042/BSR20190206

**Published:** 2019-05-31

**Authors:** Hirono Iriuchishima, Akito Maeshima, Shunsuke Takahashi, Takuma Ishizaki, Akihiko Yokohama, Norifumi Tsukamoto, Takayuki Saitoh, Hirokazu Murakami, Hiroshi Handa

**Affiliations:** 1 Department of Hematology, Gunma University Graduate School of Medicine, Gunma, Japan; 2 Division of Nephrology, Department of Internal Medicine, Jichi Medical University, Tochigi, Japan; 3 Department of Nephrology and Rheumatology, Gunma University Graduate School of Medicine, Gunma, Japan; 4 Department of Blood Transfusion, Gunma University Hospital, Gunma, Japan; 5 Department of Oncology Center, Gunma University Hospital, Gunma, Japan; 6 Department of Laboratory Sciences, Gunma University Graduate School of Health Sciences, Gunma, Japan

**Keywords:** activin A, biomarker, multiple myeloma, renal impairment

## Abstract

Renal impairment (RI) is a common complication of multiple myeloma (MM) that significantly affects treatment efficacy and mortality. However, no useful biomarkers for early detection of renal damage in MM exist. Reports indicate that activin A, a multifunctional cytokine of the TGF-β superfamily, is involved in the development and progression of various kidney diseases. In the present study, we measured urinary activin A levels in patients with newly diagnosed MM (NDMM) (*n*=41), smoldering MM (SMM) (*n*=10), and monoclonal gammopathy of undetermined significance (MGUS) (*n*=28), including monoclonal gammopathy of renal significance (MGRS), and assessed the correlation between urinary activin A and several clinical parameters. Urinary activin A, undetectable in healthy volunteers, was significantly increased in NDMM patients but not in patients with SMM and MGUS (97.3, 25.0, and 6.61 mg/gCr, respectively, *P*<0.05). In all patients with NDMM, urinary activin A levels were significantly reduced after initial treatment regardless of the therapy regimen. There was a significant correlation of urinary activin A with spot urinary protein level (*P*<0.001) and serum M-protein (*P*=0.029) but not with estimated glomerular filtration rate (eGFR), serum creatinine (Cr), N-acetyl-glucosaminidase (NAG), and serum activin A level. Histological analysis using renal biopsy samples revealed that activin A, which was absent from normal kidneys, was detected in the renal tubular cells of patients with MGRS. These data suggest that urinary activin A reflects tubular injury in MM and might aid the early detection of RI in plasma cell neoplasms.

## Introduction

Renal impairment (RI) is a common feature of multiple myeloma (MM) that complicates approximately 20–40% of newly diagnosed MM (NDMM) cases [1,2]. The most common RI is immunoglobulin-mediated, and may be caused by cast nephropathy [3], monoclonal immunoglobulin deposition disease [4], and light-chain amyloidosis [5]. RI may also be a result of other factors such as hypercalcemia, dehydration, and nephrotoxic drug use [6], and less frequently, cryoglobulinemic glomerulonephritis, proliferative glomerulonephritis, and myeloma cell infiltration [6,7].

During the last decade, the emergence of novel agents has improved the prognosis of MM patients with RI. However, their prognosis remains inferior to that of patients with normal renal function at diagnosis [8]. RI in MM is conventionally defined as a serum creatinine (Cr) level > 2.00 mg/dl or Cr clearance (Ccr) < 60 ml/min [9–11]. Recently, a cutoff of Ccr < 40 ml/min was proposed that has been widely used [12]. Nevertheless, these parameters are not sensitive in detecting the early stage of RI, which often results in delayed diagnosis or treatment. Recently, it was reported that monoclonal gammopathy of renal significance (MGRS), which represents all renal disorders caused by a monoclonal immunoglobulin secreted by a B cell or plasma cell clone, is associated with high morbidity [13]. Moreover, early treatment targeting the causal B cell or plasma cell clone when renal function is still preserved is crucial [14]. Therefore, it is important to identify biomarkers that enable the detection of early-stage RI.

Activin is a multifunctional cytokine belonging to the TGF-β superfamily that regulates the growth and differentiation of cells in various organs [15]. Its action is modulated by an endogenous activin antagonist, follistatin [16]. It has been reported that activin A negatively regulates tubular regeneration after acute kidney injury (AKI), and potently promotes renal fibrosis in unilateral-ureteral-obstruction-model rats [17–21]. Recent studies have identified that urinary activin A serves as a sensitive biomarker reflecting AKI severity [22]. However, it is unknown whether activin A is involved in renal damage in MM and whether urinary activin A is detectable in patients with MM. Consequently, our study aimed to measure urinary activin A levels in patients with MM and to identify correlations between urinary activin A levels and several clinical parameters.

## Methods

### Patients

A total of 41 patients with NDMM, 10 with asymptomatic (smoldering) MM (SMM), and 28 with monoclonal gammopathy of undetermined significance (MGUS), including MGRS, who were diagnosed at the Gunma University Hospital between October 2012 and September 2017, were included in the present study. Serum, urine, and renal biopsy samples were used for analysis. Urine from ten healthy volunteers was used as the healthy control (HC). One patient with SMM on dialysis because of chronic kidney disease was excluded. Clinical data at the time of diagnosis, including age, gender, serum and urine biochemical parameters, complete blood count, M-protein levels, clinical stages [International Staging System (ISS) stage], and bone disease, were used for analysis. The present study was approved by the Local Institutional Review Board (approval number: 855), and written informed consent was obtained from all patients.

### ELISA

We quantitated serum and urinary activin A levels using ELISA according to the manufacturer’s instructions (R&D systems, Minneapolis, MN). To smooth wide fluctuations in urinary protein concentration over the day, the measurement of the protein:creatinine ratio in single urine specimens is commonly utilized instead of 24-h urine collections. For the same reason, we used urinary activin level adjusted by urinary Cr level as a parameter in the present study.

### Immunostaining for activin A

Indirect fluorescent immunostaining was performed as described previously [23]. Briefly, the optimal cutting temperature-embedded frozen kidney sections were fixed in ice-cold methanol–acetone (1:1) for 10 min and incubated with rabbit anti-inhibin β A antibody (ab97705) (Abcam, Cambridge, U.K.) overnight at 4°C. After washing with phosphate-buffered saline, the sections were incubated with fluorescein-labeled secondary antibodies (Alexa; Molecular Probes, Eugene, OR), fluorescein lotus tetragonolobus lectin (FL-1321) from Vector Laboratories (Burlingame, CA), and 4′,6′-diamidino-2′-phenylindole dihydrochloride. Fluorescent images were recorded with the BZ-X700 all-in-one fluorescence microscope (KEYENSE, Osaka, Japan). For the immunostaining control, the primary antibody was replaced with phosphate-buffered saline, which did not show positive staining, confirming specificity.

### Statistical analysis

Statistical analysis was performed using Stat Flex version 6 (Artech Co., Ltd., Osaka). Differences between means and independence were compared using the chi-square test or Tukey’s test. The significance of differences between means was compared using a *t*test or Mann–Whitney U-test. Multiple comparisons were analyzed with the Dunn’s test. Correlation was analyzed with the Spearman rank method. *P*-values of <0.05 were considered significant.

## Results

### Patient characteristics

The patient characteristics are shown in Table 1. The study comprised 41 male and 38 female patients (median age: 68 years). More than 50% of patient had advanced-stage NDMM (ISS stage = 3). The M-protein included IgG in 49 patients (62%), Bence Jones protein in 8 (10%), and IgA in 17 (22%). Anemia, defined as hemoglobin < 10 g/dl, was diagnosed in 51% of NDMM patients. Elevated levels of serum lactate dehydrogenase were noted in 15% of all patients. Elevated levels of serum Cr (Cr > 2.00 mg/dl) were noted in 25% of all patients and in 29% of NDMM patients. Declines in the estimated glomerular filtration rate (eGFR) were noted in 27% of all patients and in 39% of NDMM patients. Lytic bone lesions were noted in 71% of NDMM patients.

**Table 1:** Baseline characteristics of the patients

Parameters	Overall	NDMM	SMM	MGUS	*P*-value
Number of patients (%)	79	41 (52)	10 (13)	28 (35)	
Male/female	41/38	23/18	4/6	14/14	0.64^1^
Age (years)					
Median (range)	68 (41–91)	69 (41–84)	62 (42–80)	68 (48–91)	n.s.^3^
MM stage, *n* (%), total	51	41	10		
ISS stage I	14/51 (27)	8/41 (20)	6/10 (60)		
Stage II	13/51 (25)	10/41 (24)	3/10 (30)		0.014^2^
Stage III	24/51 (47)	23/41 (56)	1/10 (10)		
Immunophenotype, *n* (%), total	79	41	10	28	
IgG	49/79 (62)	22/41 (54)	7/10 (70)	20/28 (71)	
IgA	17/79 (22)	10/41 (24)	3/10 (30)	4/28 (14)	0.49^1^
BJP	8/79 (10)	6/41 (15)	0/10 (0)	2/28 (7)	
Others	5/79 (6)	3/41 (7)	0/10 (0)	2/28 (7)	
Hb < 10 (g/dl), *n* (%)	25/79 (32)	21/41 (51)	2/10 (20)	2/28 (7)	0.0004^1^
Cr > 2 (mg/dl), *n* (%)	13/79 (25)	12/41 (29)	0/10 (0)	1/28 (4)	0.006^1^
eGFR > 40 ml/min, *n* (%)	21/79 (27)	16/41 (39)	0/10 (0)	5/28 (18)	0.019^1^
Serum LDH > 230 (IU/l), *n* (%)	12/79 (15)	6/41 (15)	0/10 (0)	6/28 (21)	0.27^1^
Bone lesion, *n* (%)	29/79 (37)	29/41 (71)	0/10 (0)		0.0001^2^

1 Comparison among NDMM, SMM, and MGUS groups (χ^2^ test).

2 Comparison between NDMM and SMM (χ^2^ test).

3 Comparison among NDMM, SMM, and MGUS groups (Tukey’s test).

### Elevated urinary activin A levels in NDMM patients

**Figure 1:**
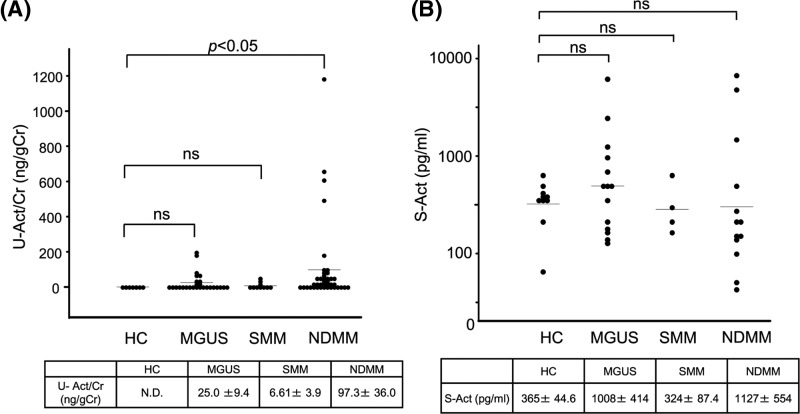
Urinary and serum activin A levels in patients with MGUS, SMM, and NDMM (**A**) Level of urinary activin A in patients with MGUS, SMM, and NDMM. The level of urinary activin A was significantly elevated in NDMM, but not in MGUS and SMM. (**B**) Level of serum activin A in patients with MGUS, SMM, and NDMM. Abbreviations: ns, not significant; S-Act, serum-activin A; U-Act/Cr, urinary-activin A/creatinine.

We first examined urinary activin A in patients with NDMM, SMM, and MGUS. Urinary activin A was not detectable in HC. In some patients with MGUS, the urinary activin A level was elevated but not significantly different compared with that in HC. Urinary activin A was not elevated in SMM but was significantly increased in NDMM (Figure 1A). We also examined serum activin A in these patients. Although several patients showed a high titer of serum activin A, there were no significant differences in serum activin A levels between these subgroups (Figure 1B).

**Figure 2:**
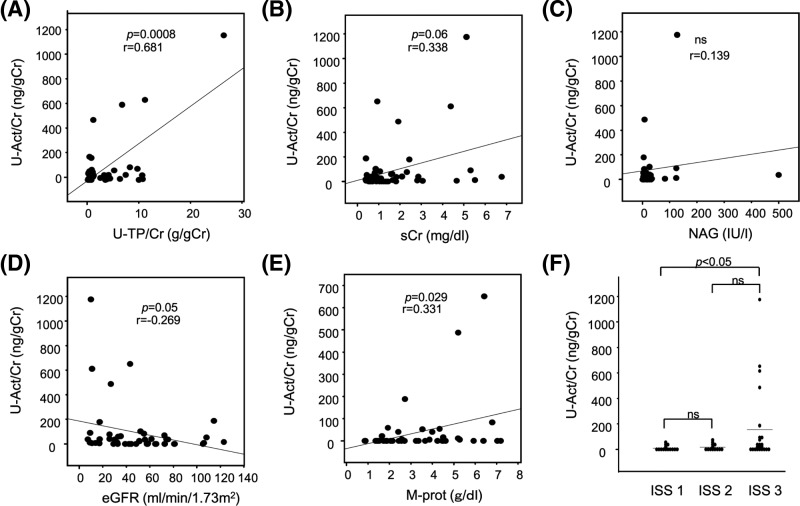
Correlation of urinary activin A level with clinical parameters (**A**–**F**) Correlation of urinary activin A level with U-TP/Cr (A), serum Cr (B), urinary NAG (C), eGFR (D), and M-protein (E). (F) Urinary activin A levels in patients at different ISS stages. Abbreviations: M-prot, M-protein; ns, not significant; U-Act/Cr, urinary-activin A/creatinine; U-TP/Cr, urinary-total protein/creatinine.

Next, we examined the correlation of urinary activin A levels with other clinical parameters, such as urinary protein level, eGFR, serum Cr, and urinary N-acetyl-glucosaminidase (NAG). In patients with proteinuria (defined by a urinary protein to Cr ratio of >0.15), urinary activin A was significantly correlated with urinary protein level (*P*=0.0008, Figure 2A), serum M-protein level (*P*=0.029, Figure 2E). Urinary activin A tended to correlate with serum Cr (Figure 2B) and eGFR (Figure 2D), but not with urinary NAG (Figure 2C). Increased levels of urinary activin A were observed in some patients without RI, defined by a serum Cr level < 2.00 mg/dl (Figure 2B) or eGFR > 40 ml/min (Figure 2D). However, no patients without elevated levels of urinary activin A had RI. When we divided the patients into three groups based on ISS stages, urinary activin A level was significantly elevated in patients at ISS stage 3 (Figure 2F).

### Marked decrease in urinary activin A level after treatment in NDMM patients

**Figure 3:**
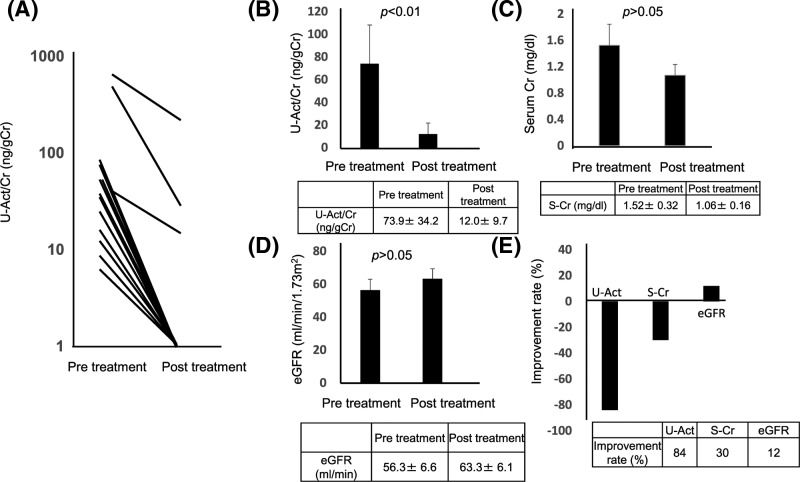
Changes in urinary activin A and serum Cr levels and eGFR after treatment (**A**) Urinary activin A levels in patients with NDMM before and after initial treatment. (**B–D**) Changes in urinary activin A (B) and serum Cr (**C**) levels and eGFR (D) after treatment. (**E**) Improvement rate of urinary activin A, serum Cr, and eGFR. Abbreviations: S-Cr, serum creatinine; U-Act/Cr, urinary-activin A/creatinine.

To analyze the impact of treatment for MM on the levels of urinary activin A, we measured the level of urinary activin A before and after initial treatment (*n*=22). The therapy regimen differed for each patient. Nonetheless, the levels of urinary activin A were significantly decreased after therapeutic intervention in all cases. Notably, urinary activin A became undetectable in all but three patients (Figure 3A). Urinary activin A was significantly decreased to undetectable levels after treatment in most of the patients (Figure 3B). Alternatively, serum Cr (Figure 3C) and eGFR (Figure 3D) levels tended to improve after treatment, but this change was not significant. The improvement rate of urinary activin A from baseline after treatment was much higher than that of serum Cr or eGFR (Figure 3E). Urinary activin A remained detectable post-treatment in three patients and the clinical responses of two patients resulted in stable disease. One of these two patients died of progressive disease 1.05 years after diagnosis. One patient achieved a stringent complete response, although his urinary activin A remained detectable. In this case, coexistent membranous glomerulonephritis possibly caused the detectable level of urinary activin A.

### Urinary activin A was not correlated with serum activin A

**Figure 4:**
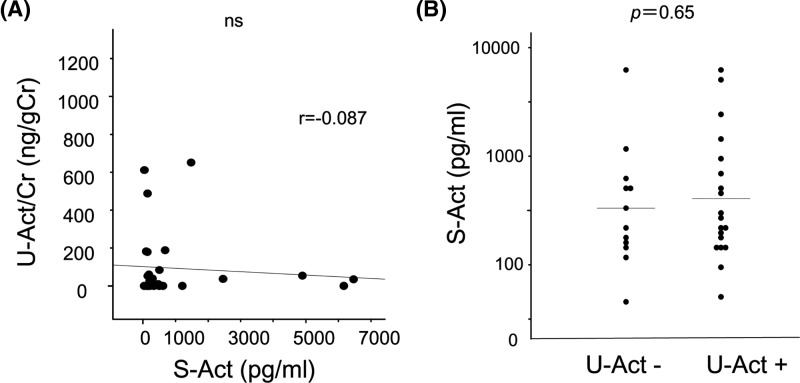
Correlation of urinary activin A level with serum activin A level (**A**) Correlation of urinary activin A level with serum activin A level. No correlation was observed between the two parameters. (**B**) Serum activin A level in patients with and without elevated urinary activin A level. Abbreviations: S-Act, serum-activin A; U-Act, urinary-activin A; U-Act/Cr, urinary-activin A/creatinine.

Next, we investigated the origin of the large amount of urinary activin A in NDMM patients. To test the possibility that urinary activin A is derived from glomerulus-filtered activin A, we compared the levels of serum and urinary activin A and found no significant correlation (Figure 4A). When we compared the level of serum activin A in patients with and without an elevation of urinary activin A, there was no significant difference between these groups (Figure 4B). These data suggest that urinary activin A does not originate from glomerulus-filtered activin A.

### No significant differences in urinary or serum activin A levels between the patients with and without bone lesions

**Figure 5:**
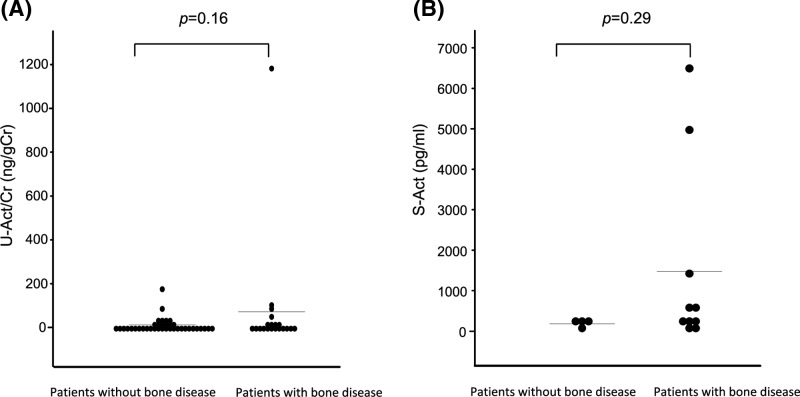
No significant differences in urinary or serum activin A levels between the patients with and without bone lesion (**A**) Urinary activin A level in patients with and without bone lesion. (**B**) Serum activin A level in patients with and without bone lesion. Abbreviations: S-Act, serum-activin A; U-Act/Cr, urinary-activin A/creatinine.

There are several reports showing that activin A has a capacity to regulate bone remodeling in MM. Malignant plasma cells induce the secretion of activin A by stromal cells, which leads to osteoblast inhibition [24]. High serum activin A was correlated with extensive bone disease and poor survival [25]. These reports prompted us to examine the correlation of urinary and serum activin A levels with the presence of osteolytic bone lesions. However, there were no significant differences in urinary activin A or serum activin A levels between the patients with and without osteolytic bone lesions (Figure 5).

### Detection of activin A in the renal tubules of patients with MGRS

**Figure 6:**
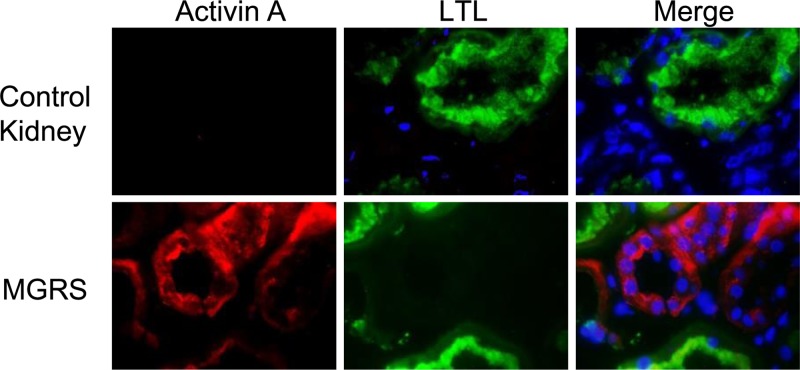
Detection of activin A in the renal tubules of patients with MGRS Immunostaining of the βA subunit of activin in kidney samples from MGRS patients and in normal kidney samples from a patient with minimal change nephrotic syndrome (control kidney). Activin A (red), fluorescein lotus tetragonolobus lectin (green), DAPI (blue). Magnification ×1000.

To elucidate the origin of urinary activin A, we performed immunostaining of available frozen biopsy samples from three MGRS patients and from one patient who had minimal change nephrotic syndrome with a histologically normal kidney (control). Activin A was undetectable in the control (Figure 6, upper panels); however, it was detected in the distal tubular cells close to lotus tetragonolobus lectin-positive proximal tubules of the kidneys in two MGRS patients (Figure 6, lower panels) and these two patients showed markedly elevated urinary activin A levels (63.1 and 179 ng/g Cr). One MGRS patient, in whom activin A was not detected in the kidney, did not show elevated urinary activin A level (0.1 ng/g Cr).

## Discussion

The present study assessed urinary activin A level and its association with RI in MM. Clinically, RI in patients with MM is defined based on serum Cr level and is included in the ‘CRAB’ (calcium level elevation, RI, anemia, and bone lesion) diagnostic criteria and the Durie and Salmon staging system of MM. However, serum Cr level varies depending on several non-renal factors, including age, sex, and muscle mass. This variation in serum Cr level and the subsequent overestimation of GFR has often led to inappropriate therapeutic interventions. Several studies on alternative biomarkers of RI in MM have recently begun to be reported [26–29]. Papassotiriou et al. [29] reported that serum neutrophil-gelatinase-associated lipocalin (NGAL) and cystatin C, which are both reliable diagnostic biomarkers of AKI and chronic kidney disease [30–35], were also useful as biomarkers of RI in patients with NDMM. Du et al. [26] demonstrated that the urinary NGAL level was significantly higher in MM patients with renal dysfunction than in MM patients without renal dysfunction, suggesting that urinary NGAL is a predictor of RI in MM.

There are at least two possible mechanisms underlying the increase in urinary activin A level in patients with NDMM. First, given that the molecular weight of activin A is 25 kDa, activin A can be theoretically filtered by glomeruli. However, urinary activin A could not be detected in HC. Similar to other urinary biomarkers including NGAL [36], glomerulus-filtered activin A might be reabsorbed by the renal tubules through endocytosis in the normal kidney. The dysfunction of tubular reabsorption might lead to the presence of activin A in the urine. The second mechanism is that urinary activin A originates from tubular cells. Activin A was detected in tubular cells of the kidneys in patients with MGRS, but not in normal kidneys (Figure 6). Tubular damage is associated with serum FLC level. Urinary activin A level was correlated with serum FLC level in our study. These data suggested that the deposition of immunoglobulins or light chains might trigger activin A expression in the tubular cells. Further study is needed to clarify this issue.

MGRS was recently delineated in 2012 [13]; however, the concept of MGRS is slightly changing. MGRS is associated with notable morbidity and even mortality due to the severity of renal and occasionally, systemic lesions induced by the monoclonal immunoglobulin [13,37–39]. Therefore, the early recognition of MGRS is crucial. In the present study, we found that urinary activin A level was increased in NDMM patients without RI and in some MGUS patients. We also found that urinary activin A level was markedly decreased after treatment in NDMM patients. The improvement in urinary activin A levels after anti-myeloma treatment was more rapid and notable compared with the improvements in serum Cr levels or eGFR.

In the clinical setting, measurement of urinary activin A level may be useful to assess early response to RI treatment in MM. These data suggest that urinary activin A serves as a sensitive biomarker reflecting the severity of RI compared with sCr and eGFR in MM.

However, the lack of long-term follow-up data in our current study prevented the assessment of the clinical applicability of urinary activin A as a predictive marker for RI in MM and MGUS. Moreover, three biopsies were not sufficient for identifying the origin of urinary activin A. Increasing the number of biopsies and the use of appropriate mouse models may help validate our hypothesis.

In conclusion, our study indicates that activin A is secreted into the urine of patients with MM and MGUS. Urinary activin A level was significantly higher in MM patients, even in those without RI, and notably decreased after treatment in NDMM patients. Activin A was present in the tubular cells of the kidney. These data suggest that urinary activin A is a promising biomarker for indicating the presence of tubular damage in patients with plasma cell dyscrasias. To verify the predictive value of urinary activin A, we are prospectively observing the renal function of patients with and without elevated urinary activin A level. Once its predictive value is verified, urinary activin A may aid in decision-making for early intervention before RI manifestation.

## Abbreviations

AKI: acute kidney injury
Ccr: creatinine clearance
Cr: creatinine
eGFR: estimated glomerular filtration rate
FLC: free light chain
HC: healthy control
ISS: International Staging System
MGRS: monoclonal gammopathy of renal significance
MGUS: monoclonal gammopathy of undetermined significance
MM: multiple myeloma
NAG: N-acetyl-glucosaminidase
NDMM: newly diagnosed MM
NGAL: neutrophil-gelatinase-associated lipocalin
RI: renal impairment
SMM: smoldering MM
TGf-β: transforming growth factor-beta
